# Sources of Mercury Exposure for U.S. Seafood Consumers: Implications for Policy

**DOI:** 10.1289/ehp.0900811

**Published:** 2009-09-29

**Authors:** Noelle E. Selin, Elsie M. Sunderland, Christopher D. Knightes, Robert P. Mason

**Affiliations:** 1 Joint Program on the Science and Policy of Global Change, Massachusetts Institute of Technology, Cambridge, Massachusetts, USA;; 2 School of Engineering and Applied Sciences, Harvard University, Cambridge, Massachusetts, USA;; 3 U.S. Environmental Protection Agency, National Exposure Research Laboratory, Athens, Georgia, USA;; 4 Department of Marine Sciences, University of Connecticut, Groton, Connecticut, USA

**Keywords:** biogeochemical cycling, exposure, mercury, seafood

## Abstract

**Background:**

Recent policies attempting to reduce adverse effects of methylmercury exposure from fish consumption in the United States have targeted reductions in anthropogenic emissions from U.S. sources.

**Objectives:**

To analyze the prospects for future North American and international emissions controls, we assessed the potential contributions of anthropogenic, historical, and natural mercury to exposure trajectories in the U.S. population over a 40-year time horizon.

**Methods:**

We used models that simulate global atmospheric chemistry (GEOS-Chem); the fate, transport, and bioaccumulation of mercury in four types of freshwater ecosystems; and mercury cycling among different ocean basins. We considered effects on mercury exposures in the U.S. population based on dietary survey information and consumption data from the sale of commercial market fish.

**Results:**

Although North American emissions controls may reduce mercury exposure by up to 50% for certain highly exposed groups such as indigenous peoples in the Northeast, the potential effects of emissions controls on populations consuming marine fish from the commercial market are less certain because of limited measurements.

**Conclusions:**

Despite uncertainties in the exposure pathway, results indicate that a combination of North American and international emissions controls with adaptation strategies is necessary to manage methylmercury risks across various demographic groups in the United States.

Mercury is a global pollutant that is ubiquitous in the environment. Once deposited into ecosystems, inorganic Hg may be converted to methylmercury (MeHg), which bioaccumulates in food webs. MeHg exposure can cause severe human health effects, including immune system suppression, neurodevelopmental delays in children, and compromised cardiovascular health in adults (Mergler et al. 2007). Human health data suggest that 300,000–600,000 U.S. children are born each year with blood MeHg levels exceeding the U.S. Environmental Protection Agency’s (EPA) reference dose (RfD) (Mahaffey et al. 2004; Trasande et al. 2005). Recent regulatory efforts to reduce MeHg exposures have focused on controlling anthropogenic sources such as waste incinerators and coal-fired utilities (U.S. EPA 2005). However, the global biogeochemical Hg cycle has been significantly altered by historical Hg releases, and the timescales required for recovery are uncertain (Lamborg et al. 2002; Mason and Sheu 2002; Selin et al. 2008; Sunderland and Mason 2007; Swain et al. 2007). We applied atmospheric, oceanic, and freshwater ecosystem fate and bioaccumulation models to illustrate the potential contributions of present-day anthropogenic, historical anthropogenic, and natural Hg to exposure trajectories in U.S. populations over a 40-year time horizon (to 2050). We used this analysis to assess the prospects for emissions reductions and to identify gaps in present understanding of the Hg exposure pathway.

Relative contributions of present-day anthropogenic sources to deposition vary considerably among locations (Cohen et al. 2004; Seigneur et al. 2004; Selin et al. 2008, Sunderland et al. 2008). Fish MeHg levels depend on atmospheric loading rates, ecosystem-specific properties, and food-web structure (Harris et al. 2007; Munthe et al. 2007). Individual seafood consumption choices also play a large role in determining exposures and resulting risks (Burger and Gochfeld 2004; Carrington et al. 2004; Stern et al. 2001). Effectively managing MeHg risks therefore requires information on the exposure pathway at both local and global scales. For example, although subsistence and recreational fishers may harvest and consume fish from local water bodies, most individuals obtain the majority of their fish from the commercial market, which combines locally harvested and imported species (Carrington et al. 2004; Sunderland 2007). Migratory pelagic marine species such as tuna and swordfish from the commercial market account for more than half of U.S. populationwide Hg intake (Sunderland 2007); thus, understanding Hg dynamics in open-ocean environments is especially important. This means that the relationships among various sources, MeHg levels in aquatic systems, bioaccumulation in food webs, and consumption patterns that result in human exposure must be analyzed at multiple spatial and temporal scales. Although many elements of this exposure pathway are uncertain, policy analysis requires synthesis of our best-available understanding to quantify these processes and to determine the effectiveness of different Hg control strategies. Environmental modeling combines disparate atmospheric, aquatic, and human health data with our best understanding of underlying processes to assist decision making (National Research Council 2007).

Previous studies have analyzed potential benefits from U.S. Hg emissions controls on exposure in parts of the U.S. population. Rice et al. (2005) analyzed both marine and freshwater exposure pathways, assuming a linear and instantaneous change in fish Hg levels with declines in atmospheric deposition. The U.S. EPA (2005) analyzed the effects of regulating emissions from coal-fired utilities on exposure of recreational fishers and their families, assuming a linear relationship between atmospheric deposition and fish MeHg. Trasande et al. (2005, 2006) analyzed the costs associated with Hg emissions from U.S. utilities by assuming a linear relationship between atmospheric deposition and fish MeHg.

To assess the prospects of policies to address Hg exposure, we go beyond these previous studies by linking the contributing source regions for atmospheric Hg emissions to temporal trends in exposure, using physically meaningful simulations of Hg fate and transport. We divide emission into natural Hg, present-day anthropogenic Hg [from North America (NA) and outside NA], and historically emitted anthropogenic Hg. We used a global three-dimensional atmospheric chemistry transport model (GEOS-Chem) to calculate the relative contribution of each emission source to present-day atmospheric Hg deposition. We explore the timescales required for each component of atmospheric deposition to cycle through ecosystems by simulating Hg transport, speciation, and bioaccumulation. To do this, we combined GEOS-Chem deposition with ecosystem-scale fate and bioaccumulation models developed by the U.S. EPA (Knightes et al. 2009) and a multicompartment global box model for oceanic Hg cycling (Sunderland and Mason 2007). We applied this methodology to illustrate how the source attribution of freshwater and marine fish MeHg may change over a 40-year time horizon. We analyzed how changes in exposure may be affected by changes in environmental concentrations resulting from emission controls for both freshwater and marine pathways using per-capita fish consumption rates (Sunderland 2007) and the consumption patterns of sensitive groups (Mahaffey et al. 2004; Moya 2004).

We divided emissions by source following Selin et al. (2008). Natural Hg sources account for approximately one-third of present global emission and deposition (Mason and Sheu 2002). Direct anthropogenic emissions from sources within and outside NA are also roughly one-third of emissions and deposition. Historical anthropogenic Hg comprises the remaining third of present-day deposition and includes the anthropogenic Hg already in aquatic systems and the reemission and cycling of anthropogenic Hg historically deposited from the atmosphere. By distinguishing among exposures to Hg from these sources, we can assess both the prospects for direct emissions reductions (decreases in the NA and non-NA anthropogenic sources) and the timescales of ecosystem response to historical anthropogenic contamination, which is not directly addressed by contemporary emissions reduction policies.

## Materials and Methods

We simulated Hg emissions, chemistry, and atmospheric deposition using the GEOS-Chem model, described in detail by Selin et al. (2007, 2008), with updates described by Selin and Jacob (2008). The GEOS-Chem simulation has been extensively evaluated against atmospheric concentration and deposition measurements and matches seasonal and spatial trends (Selin et al. 2007, 2008). GEOS-Chem simulates three atmospheric Hg species. Elemental mercury [Hg(0)], the dominant (> 95%) species, has an atmospheric lifetime of 0.5–2 years and transports globally. Divalent and particulate Hg species [Hg(II)_(g,aq)_ and Hg(P), respectively] are shorter lived (lifetime of days to weeks) and deposit closer to sources.

Direct anthropogenic releases are mainly from coal-fired utilities, metal smelting, mining, and waste incineration (Pacyna et al. 2006). GEOS-Chem anthropogenic emissions are from the year 2000 global inventory (Pacyna et al. 2006), modified as described in Selin et al. (2008) to satisfy observational constraints. Total Hg emission in the model is 11,200 Mg/year, of which 3,400 Mg/year is from direct anthropogenic sources. Globally, 58% of direct anthropogenic emissions are Hg(0), 33% are Hg(II), and 9% are Hg(P). Asia contributes approximately 54% of global anthropogenic Hg emissions, and NA contributes approximately 7% (Pacyna et al. 2006). Land and ocean emissions, all as Hg(0), of preindustrial and historical anthropogenic origin are from the coupled land-ocean-atmosphere simulation described by Selin et al. (2008).

GEOS-Chem simulates wet and dry deposition of Hg(II) and Hg(P) and dry deposition of Hg(0) (Selin and Jacob 2008). In GEOS-Chem, 34% of Hg(II)/Hg(P) deposits to land and 67% to oceans (Selin et al. 2008). Here, we used wet and dry deposition of Hg(II) and Hg(P), the predominant forms of atmospheric loading to aquatic ecosystems. [Although Hg(0) represents approximately 30% of deposition, it is subject to rapid two-way exchange between the atmosphere and surface.] Globally, the mean percentage of total deposition from a source is equivalent to its contribution to emission. However, the percentage varies spatially; contributions of emissions sources to deposition in a particular location depend on both the species of Hg emitted and atmospheric chemical processes, transport, and circulation patterns. Annual mean contribution percentages by source do not change significantly based on the choice of particular meteorologic year (Selin and Jacob 2008; Selin et al. 2007). Our results are based on meteorologic data for 2004–2005.

Table 1 shows GEOS-Chem deposition and source contributions for two regional deposition scenarios and for global ocean basins. Contributions to deposition from natural sources are from the preindustrial simulation described in Selin et al. (2008). Deposition from emissions within and outside of NA is determined from simulations with NA and total anthropogenic emission sources shut off, respectively. We calculated historical anthropogenic sources by the difference between summed natural and direct anthropogenic deposition and total deposition. For freshwater ecosystems, we used two deposition scenarios: scenario 1 and 2, with deposition characteristics consistent with the U.S. Northeast and Southeast regions, respectively. We chose these scenarios because previous analyses have shown that Hg deposition in these regions comes from different source combinations. In scenario 1, based on the Northeast (40–44° N, 72.5–77.5° W), GEOS-Chem attributes 59% of deposition to NA anthropogenic sources, 9% to anthropogenic sources outside NA, 16% to natural sources, and 16% to historical anthropogenic Hg (Selin and Jacob 2008). In scenario 2, based on the Southeast (24–28° N, 77.5–82.5° W), GEOS-Chem attributes only 11% to NA anthropogenic sources, although measured wet deposition in this region is the highest in the United States (National Atmospheric Deposition Program 2009). The remainder comes from anthropogenic sources outside NA (23%), natural sources (42%), and historical anthropogenic Hg (24%) (Selin and Jacob 2008).

Uncertainties are inherent in any effort to model global Hg fate and transport, including specifying the atmospheric redox chemistry of Hg(0)/Hg(II) and quantifying Hg fluxes between the atmosphere and surface (Lin et al. 2006; Pongprueksa et al. 2008). Although the influence of these uncertainties on our results is difficult to quantify, source contributions in GEOS-Chem are similar to other global Hg models. Seigneur et al. (2004) report that NA emissions contribute on average 25–32% to U.S. deposition and are highest near sources in the Midwest and lowest in the Southeast.

Across ocean basins, the mean percentage of deposition from present-day anthropogenic sources varies between 23% and 35%. For each ocean basin, we calculated deposition over the area corresponding to the Sunderland and Mason (2007) model compartments, with the exception of the Mediterranean Sea. For the Mediterranean, we scaled GEOS-Chem deposition fractions to empirical estimates from Sunderland and Mason (2007). The 4° × 5° GEOS-Chem resolution does not distinguish the Mediterranean from desert regions characterized by large-scale subtropical downwelling and thus predicts higher than expected deposition based on observational data (Kotnik et al. 2007).

Sunderland and Mason (2007) used deposition from three atmospheric models to assess the influence of deposition uncertainty on ocean concentration trajectories over the next several decades. Differences between atmospheric deposition rates from GEOS-Chem and other models are most apparent for the Atlantic Ocean, where GEOS-Chem deposition results in slight concentration increases over the next several decades, compared with decreases for other atmospheric models. Additional data on concentration trends in different ocean basins are needed to resolve this uncertainty. However, relative contributions of various sources to oceans are less uncertain than overall trajectories, because they reflect relative magnitudes of global emission.

We used contemporary atmospheric deposition rates and present-day fractions of deposition from each source category as input to the aquatic Hg fate and transport models. Future emissions scenarios are not presently available for global-scale Hg cycling applications. Our analysis helps to illuminate the potential magnitude and timing of ecosystem responses, but we advise caution when interpreting results for future exposure pathways. Our approach may have underestimated contributions from Asia, which increased Hg emissions approximately 70% between 1990 and 2000 (Pacyna et al. 2006) and is rapidly increasing its energy use, and overestimated contributions from NA and Europe due to decreased emissions. We focused our analysis on Hg concentration trajectories in freshwater and marine ecosystems over a 40-year time frame (roughly 2050), a medium to long time horizon in policy making. Global emissions have been relatively constant for the past decade, although current estimates project likely increases by 2050 (Streets et al. 2009). Our analyses could be further refined as additional information on historical and future Hg emissions and trends in environmental concentrations become available (Mason et al. 2005; Streets et al. 2009).

### Freshwater modeling

We combined GEOS-Chem deposition with previously published watershed, water body, and food web bioaccumulation Hg models for four different types of freshwater ecosystems (Knightes et al. 2009). Each of these modeling frameworks is available for review (U.S. EPA 2009) and has been extensively evaluated (Ambrose et al. 2005; Brown et al. 2007; Knightes 2008). We simulate Hg dynamics in water bodies using the spreadsheet-based Ecological Risk Assessment for the Fate of Mercury (SERAFM) model and the Water Quality Analysis Simulation Program (WASP) (Knightes 2008; U.S. EPA 1997a). Watershed-Hg dynamics are based on the U.S. EPA Region 4 Watershed Characterization System Mercury Loading Model and land-cover characteristics (Greenfield et al. 2002; Knightes et al. 2009). Bioaccumulation is simulated using the Bioaccumulation and Aquatic System Simulator (BASS) (Barber 2006).

The four ecosystems we consider here have biological and geochemical properties consistent with a seepage lake, coastal plain river, drainage lake, and stratified lake. We refer to these as ecosystems A–D, respectively, recognizing that responses within each class of freshwater ecosystems can vary substantially depending on a variety of biogeochemical attributes. Simulations driven with atmospheric deposition from GEOS-Chem were initially calibrated to the specific ecologic characteristics described by Knightes et al. (2009). The models use empirically constrained, first-order and pseudo-first-order rate constants to simulate transformations among Hg species [MeHg, Hg(II), and Hg(0)] through methylation, demethylation, oxidation, and reduction. The BASS model simulates MeHg trophic dynamics from uptake at the base of the food web to top predator fish species. Algorithms used to describe Hg speciation, transport, and bioaccumulation in these models and model evaluation are described elsewhere (Barber 2006; Knightes 2008; Knightes et al. 2009).

For each ecosystem, models are initialized with empirically constrained concentrations (Knightes et al. 2009) and run with present-day deposition from GEOS-Chem for scenarios 1 and 2 (Table 1). For the watershed-dominated ecosystem B, an initialization period (50 years) is needed because of a slower response time (Knightes et al. 2009). Because geochemical characteristics affecting Hg speciation have changed over time, we cannot simulate the true preindustrial ecosystem state. Instead, we simulate the combined historical anthropogenic and natural components of deposition. This combined fraction comprises all Hg released before the present day regardless of source. The change in this fraction over time is determined by the difference between a simulation with total deposition and those with direct anthropogenic sources only. For consistency, we compare the temporal responses across ecosystems using a trophic level 4 fish species.

### Oceanic modeling

The Sunderland and Mason (2007) model uses a simplified physical framework for ocean circulation that approximately matches the limited oceanic Hg data available. The model is driven by atmospheric deposition but also includes Hg transport associated with lateral and vertical circulation, settling particulate matter, evasion of Hg(0), and freshwater discharges. Here, we focus on water column depths that are likely to be the most relevant for MeHg production, transport, and biological exposures (Choy et al. 2009; Sunderland 2007; Sunderland et al. 2009).

To assess the contributions of different emission sources to ocean concentrations, we first ran the ocean model with total present-day deposition. The evolution over time of ocean basin concentrations from anthropogenic emissions within and outside of NA is diagnosed by running the model with GEOS-Chem deposition with those sources shut off. We calculated the natural component by running the ocean model with GEOS-Chem preindustrial deposition. The historical anthropogenic component is the difference between the total concentration and the sum of the natural and direct anthropogenic components.

## Results

### Temporal trends in freshwater fish Hg and associated exposure

**Figure 1:**
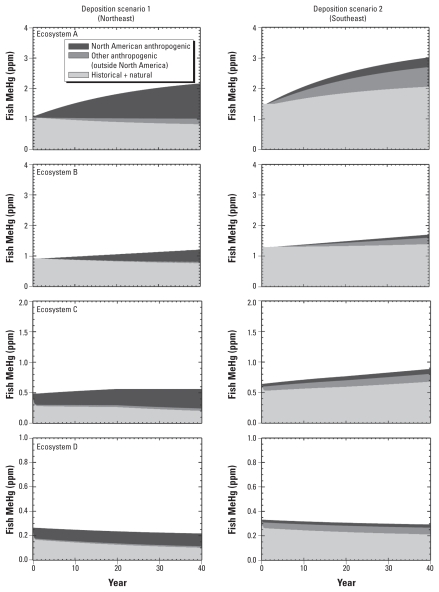
Temporal evolution of fish MeHg sources for model ecosystems A–D to U.S. deposition scenario 1 (Northeast) and scenario 2 (Southeast; see Table 1). Note difference in scale for ecosystems C and D. The model is run for 50 years; the first 10 years are treated as initialization.

Figure 1 shows the temporal evolution of MeHg sources in predatory fish for the four model ecosystems for the Northeast and Southeast deposition scenarios. Our results show that the fraction of fish MeHg from NA anthropogenic sources varies considerably both among systems and between the two deposition scenarios after 40 years of constant atmospheric loading. In the model ecosystems, initial empirically constrained concentrations are not at steady state with respect to deposition inputs. Where concentrations are increasing (decreasing), this suggests that historical loadings to our hypothetical ecosystems were less than (greater than) simulated deposition. Recent analysis suggests that deposition in the U.S. Northeast has been declining, whereas Southeast deposition shows no trend (Butler et al. 2008).

Differences in fish MeHg response times are due to ecosystem-specific factors such as evasion rates, sediment burial rates, and active sediment layer depths (Knightes et al. 2009). Although NA sources contribute more than half of deposition to all ecosystem types for scenario 1, their contribution at year 40 to fish MeHg ranges from 40% (ecosystem B) to 60% (ecosystem C). In scenario 2, the contribution from NA sources ranges from 7% to 11% after 40 years. Sources outside NA contribute a larger fraction of MeHg in scenario 2 than in scenario 1, reflecting their greater contribution to deposition.

The change in historical plus natural loading over time is more complex. In some systems, such as ecosystem A in scenario 2, the historical plus natural contribution increases over time, whereas in others, such as ecosystem D, it decreases. Decreases in the relative historical contribution reflect the rapid turnover of Hg deposited in the few years before *t* = 0, whereas increases reflect both the influence of watersheds (ecosystem B) and the increases of initial concentrations to reflect a steady state relative to the deposition from historical sources (ecosystem A). In all systems, in the very long term, the source signature in fish will approach that of deposition. However, these timescales may approach the timescales in which changes in Hg loading affects emissions of anthropogenic Hg that is stored from earlier deposition to soils and oceans (~100 years). At present, stored Hg is increasing in response to anthropogenic loadings. If storage continues to increase, deposition from historical sources will also increase.

**Figure 2:**
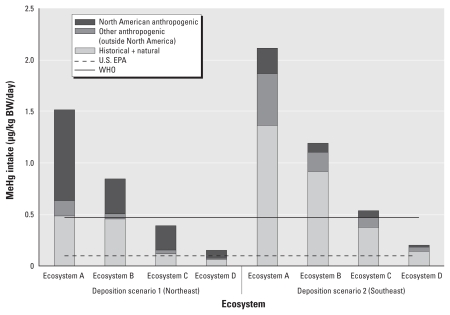
MeHg intake source attribution for a hypothetical consumer eating 0.7 g fish/kg body weight per day for the four model ecosystems under deposition scenarios 1 and 2. The solid line indicates the WHO maximum intake recommendation; the dashed line shows the U.S. EPA RfD.

To illustrate the impacts of changes in freshwater fish Hg on human exposure, we assess the range of possible benefits of decreases in emissions for hypothetical consumers of fish from these ecosystems, using the variation among source contributions to regions and ecosystems. We apply these source contributions to the lower-bound mean adult Native American fish intake (0.7 g/kg body weight (BW) per day) reported in Moya (2004). Figure 2 shows the sources of MeHg intake (micrograms per kilogram of BW per day) for a consumer eating trophic level 4 fish from ecosystems A–D at year 40, for both deposition scenarios. Figure 2 shows MeHg intake for a consumer eating 0.7 g fish/kg BW/day, corresponding to approximately 40–50 g/day for a 60 to 70 kg person. Also shown is the World Health Organization (WHO) maximum intake criterion (0.47 μg Hg/kg BW/Hg/day) and the U.S. EPA MeHg RfD (0.1 μg Hg/kg BW/day). The NA contribution represents the fraction of intake that can be affected by present and future NA policy alone; the other anthropogenic contribution by policies outside NA and the historical plus natural contribution represent the intake that cannot be altered by Hg-specific emission control policies. Estimated MeHg intake by source for mean and 95th percentile fish intake for recreational freshwater anglers (8 and 25 g/day, respectively) and Native American subsistence fishers (70 and 170 g/day) (U.S. EPA 1997b) are presented in the Supplemental Material available online (doi:10.1289/ehp.0900811.S1 via http://dx.doi.org/).

The potential effectiveness of NA regulations alone varies substantially across the different ecosystems and the two deposition scenarios. For the hypothetical consumer in Figure 2, MeHg intake exceeds WHO guidelines for five ecosystems, whereas the EPA RfD is exceeded for all eight. Emissions reductions in NA will result in significant decreases in MeHg intake, especially under scenario 1 and in faster responding ecosystems like ecosystem A. For scenario 2, emissions controls outside NA are as important as NA controls.

However, NA reductions alone will in most cases not result in decreases below guideline levels. Our results suggest that historical emissions will continue in the long term to contribute MeHg in excess of exposure guidelines, particularly in areas with deposition similar to scenario 2, such as the U.S. Southeast. The only long-term sink for Hg in the global biogeochemical cycle is sediment burial. Historical anthropogenic Hg continues to be emitted to the atmosphere from global soils and oceans, which have been enriched over time, and will continue to deposit to ecosystems until concentrations return to their steady-state levels (on timescales that can range from centuries to millennia).

### Temporal trends in oceanic Hg and exposure from marine fish

Although Hg exposure from freshwater ecosystems is important for highly exposed groups (e.g., recreational and subsistence fishers), for the average U.S. individual most fish consumed comes from the commercial market. Previous studies have shown that > 90% of the edible species sold in the commercial market are from marine and estuarine systems (Carrington et al. 2004; Sunderland 2007). Thus, on the populationwide level, most MeHg exposure is from estuarine and marine seafood. Further, > 70% of Hg exposure from seafood sold in the U.S. commercial market is from fish caught beyond the 200 mile limit for U.S. domestic waters, and less than half of the domestic catch is harvested within 3 miles from shore (National Marine Fisheries Service 2007; Sunderland 2007). To analyze trends in exposure from these sources, we need information that links atmospheric deposition, concentrations of MeHg in coastal and open ocean environments, marine fish Hg levels, and local consumption data.

Presently no modeling framework exists that links atmospheric fate and transport of inorganic Hg to oceanic MeHg concentrations and bioaccumulation in marine fish. This represents a major gap in our capability to analyze the effects of Hg emissions reductions on human exposures from marine fish. However, existing models allow us to link sources of deposition to total Hg concentrations in seawater as a first step toward such an analysis.

**Figure 3:**
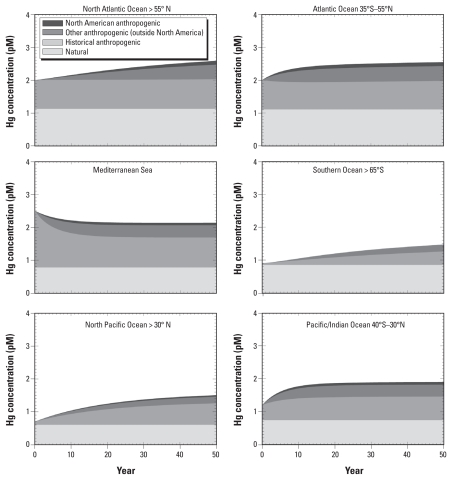
Evolution of relative contributions of anthropogenic (NA and outside NA), historical anthropogenic, and natural sources to Hg concentrations in six surface ocean basins. Abbreviations: N, north; S, south.

Figure 3 shows the evolution over time of concentrations in eight surface and intermediate ocean basins attributed to various sources. Present and future emissions begin to accumulate in each ocean basin at time *t* = 0. Over time, the Hg concentration in ocean basins approaches steady state, where the percentage from each source equals its percentage in deposition.

As shown in Figure 3, present and future contributions to ocean concentrations after 40 years are still lower than their percentages in deposition (Table 1). Total Hg concentrations in the top 300–1500 m of the water column simulated here require decades to centuries to reach steady state with contemporary atmospheric deposition. The fraction of seawater Hg attributed to 40 years of present-day deposition ranges from 14% to 23%. The highest values are in the Pacific Ocean at mid-latitudes (below 30° N), and the lowest in the Southern Ocean. Historical anthropogenic contributions after 40 years range from a low of 26% in the Southern Ocean to a high of 42% in the Mediterranean Sea. The growth of the historical contribution to concentrations occurs in part because historical anthropogenic Hg is a continuing fraction of Hg deposition into the future, due to its continued cycling in the environment. The temporal response of total Hg concentrations in the ocean surface mixed layer (< 100 m) will be relatively rapid (several years) compared with those of intermediate waters (decades to centuries) and deep waters (many centuries) (Sunderland and Mason 2007; Sunderland et al. 2009). Temporal dynamics of MeHg in oceans may differ from dynamics of total Hg because water column methylation processes occur primarily in the low-oxygen regions below the well-mixed surface layer (Mason and Fitzgerald 1990; Mason and Sullivan 1999; Mason et al. 1998; Sunderland et al. 2009).

## Discussion

Our results show that the potential impact of NA and global emissions reductions on Hg exposure varies considerably for different fish-eating populations. Emissions controls in NA can substantially reduce exposure of freshwater fish consumers in areas dominated by NA deposition (e.g., U.S. Northeast), especially those who harvest fish from rapidly responding lakes dominated by direct atmospheric deposition to the water surface and with low ratios of watershed to water surface area (Knightes et al. 2009). In other regions with deposition similar to the U.S. Southeast, deposition from sources outside NA is as important as that from NA sources. Thus, emission reductions outside NA are likely very important for reducing fish Hg burdens and human exposure for freshwater fish consumers in this region. Sources outside NA are also likely to dominate future Hg exposure trends from fish sold in the U.S. commercial market. Our analysis highlights that historical anthropogenic Hg will have a continuing, long-term impact on exposure regardless of Hg emissions. Reducing present-day emissions has the dual benefit of reducing future exposures from direct and reemitted Hg in the environment.

Our open-ocean modeling results show that NA emissions sources comprise a relatively limited fraction of seawater total Hg over the next 40 years (Figure 3). Sparse measurements of MeHg concentrations in marine ecosystems limit our ability to relate atmospheric Hg deposition changes to MeHg levels in marine fish (Sunderland et al. 2009). Recent studies have reinforced the importance of the marine water column for MeHg production and Hg bioavailability in open ocean environments (Cossa et al. 2009; Kirk et al. 2008; Sunderland et al. 2009). To approximate temporal trajectories in exposure from pelagic marine fish, we assume that over long timescales changes in inorganic Hg concentrations will be reflected by differences in oceanic MeHg and subsequent bioaccumulation in pelagic marine fish. Although this approach improves on previous studies that do not consider temporal trends in Hg concentrations as a function of ocean circulation and other loss processes (Rice et al. 2005; Trasande et al. 2005), further research is needed to resolve the relative importance of MeHg production in productive ocean margin regions for Hg levels in some marine fish species (Hammerschmidt and Fitzgerald 2006; Hollweg et al. 2009). Our results suggest that NA regulatory actions alone will not be sufficient to reduce Hg concentrations in pelagic marine species due to large contributions to seawater Hg from sources outside NA (Figure 3). Because Hg concentrations in much of the ocean have not yet reached steady state with contemporary atmospheric deposition (Sunderland and Mason 2007; Sunderland et al. 2009), U.S. individual Hg exposures from commercial market fish are likely to continue to rise over the next several decades. Furthermore, global Hg sources are likely to increase, especially in Asia (50% of present day global emissions), as growing economies use more coal-based energy. Substantial global reductions in emissions are therefore needed to prevent further increases in concentrations in pelagic marine fish and associated human exposure.

The potential for emissions reductions beyond NA to reduce exposure, as presented here, should be viewed as a lower bound. Emissions under a “business-as-usual” scenario are likely to increase (particularly in Asia) rather than stay constant. Streets et al. (2009) project changes in global Hg emissions under different Intergovernmental Panel on Climate Change (IPCC) scenarios between 96% and − 4%; they note that developments in Asia largely dictate the variation across scenarios. Emissions reductions within and beyond NA will furthermore have an additive effect. The methodology presented here could be applied to specific emissions scenarios, ecosystems, and exposure patterns to project future trends in exposure.

A substantial long-term benefit of anthropogenic emissions reductions, not directly considered in our analysis, is the avoided emissions that would have added to the pool of historical anthropogenic Hg, which cycles over very long timescales in the environment and contributes to exposure. Thus, future increases enhance deposition not only in the short term but also in the long term because of global cycling. This would be avoided with reduction in emissions now.

Minimizing human Hg exposure will require both mitigation and adaptation because of the long-term impacts of historical Hg. In this way, the Hg cycle’s effect on continuing exposure is similar to the carbon cycle influence on climate change. Because of carbon cycle timescales, anthropogenic warming and sea level rise would continue for centuries even if greenhouse gas concentrations stabilized (IPCC 2007). In response to this, policy actions have begun to focus both on mitigation and adaptation (Pielke 1998). In the case of Hg, policy makers have already initiated some adaptation measures, such as dietary advice for pregnant women and children on limiting their Hg intake (U.S. Department of Health and Human Services and U.S. EPA 2004). The long-term nature of the Hg problem suggests that a variety of approaches, at different scales, is necessary (Selin and Selin 2006).

## Figures and Tables

**Table 1:** GEOS-Chem deposition estimates (μg/m^2^ per year) and percentage of total deposition, by emission source, for ocean basins and U.S. regions, used as input to ecosystem and ocean models.

Scenario/region	Total deposition	Natural	NA anthropogenic	Other anthropogenic (outside NA)	Historical anthropogenic
Scenario 1 (U.S. Northeast, 40–44° N, 77.5–82.5° W)	24	4.0 (16%)	14 (59%)	2.1 (9%)	3.9 (16%)
Scenario 2 (U.S. Southeast, 24–28° N, 77.5–82.5° W)	34	14 (42%)	3.7 (11%)	7.9 (23%)	8.2 (24%)
North Atlantic Ocean (> 55° N)	9.7	3.4 (35%)	0.7 (7%)	2.7 (28%)	3.0 (30%)
Southern Ocean (> 65° S)	1.4	0.4 (29%)	0.1 (6%)	0.2 (17%)	0.7 (47%)
Pacific/Indian Ocean (40° S to 30° N)	20	6.2 (31%)	0.9 (4%)	4.0 (20%)	8.7 (44%)
North Pacific Ocean (> 30° N)	15	3.8 (25%)	0.7 (5%)	3.2 (21%)	7.6 (50%)
Atlantic Ocean (35° S to 55° N)	20	6.7 (34%)	1.1 (6%)	4.2 (21%)	7.9 (40%)
Mediterranean Sea	27	8.1 (30%)	1.1 (4%)	5.7 (21%)	12 (45%)

Abbreviation: N, North; NA, North America; S, South; W, West. Totals may not be exact due to rounding.
